# Preliminary Study of Preheated Decarburized Activated Coal Gangue-Based Cemented Paste Backfill Material

**DOI:** 10.3390/ma16062354

**Published:** 2023-03-15

**Authors:** Renlong Tang, Bingchao Zhao, Chuang Tian, Baowa Xu, Longqing Li, Xiaoping Shao, Wuang Ren

**Affiliations:** 1 Energy School, Xi’an University of Science and Technology, Xi’an 710054, China; 2 Key Laboratory of Western Mines and Hazards Prevention, Ministry of Education of China, Xi’an 710054, China; 3 School of Architecture and Civil Engineering, Xi’an University of Science and Technology, Xi’an 710054, China

**Keywords:** preheated decarburized, backfill, coal gangue, compressive strength, microstructure, leaching

## Abstract

This study proposes a novel idea of the use of coal gangue (CG) activation and preheated decarburized activated coal CG-based cemented paste backfill material (PCCPB) to realize green mining. PCCPB was prepared with preheated decarburized coal CG (PCG), FA, activator, low-dose cement, and water. This idea realized scale disposal and resource utilization of coal CG solid waste. Decarbonization and activation of CG crushed the material to less than 8 mm by preheated combustion technology at a combustion temperature of 900 °C and a decarbonization activation time of 4 min. The mechanism of the effect of different Na_2_SO_4_ dosages on the performance of PCCPB was investigated using comprehensive tests (including mechanical property tests, microscopic tests, and leaching toxicity tests). The results show that the uniaxial compressive strength (UCS) of C-S2, C-S3, and C-S4 can meet the requirements of backfill mining, among which the UCS of C-S3 with a curing time of 3 d and 28 d were 0.545 MPa and 4.312 MPa, respectively. Na_2_SO_4_ excites PCCPB at different curing time, and the UCS of PCCPB increases and then decreases with the increase in Na_2_SO_4_ dosage, and 3% of Na_2_SO_4_ had the best excitation effect on the late strength (28 d) of PCCPB. All groups’ (control and CS1-CS4 groups) leachate heavy metal ions met the requirements of groundwater class III standard, and PCCPB had a positive effect on the stabilization/coagulation of heavy metal ions (Mn, Zn, As, Cd, Hg, Pb, Cr, Ba, Se, Mo, and Co). Finally, the microstructure of PCCPB was analyzed using FTIR, TG/DTG, XRD, and SEM. The research is of great significance to promote the resource utilization of coal CG residual carbon and realize the sustainable consumption of coal CG activation on a large scale.

## 1. Introduction

One of China’s major strategies is sustainable development, and for the coal industry to develop sustainably, reasonable disposal of coal gangue solid waste is necessary. Coal is the ballast stone of China’s energy security, but the coal mining process also produces a series of environmental problems, such as surface damage, coal gangue (CG) emissions, etc. [1,2]. CG accounts for 10–25% of the total coal mining [3], and over the years, China has accumulated more than 7 billion tons of CG solid waste and a growth rate of 150 million t/a [4]. CG stockpiles occupy a large number of land resources and also have potential environmental hazards such as spontaneous combustion and the leaching of heavy metal ions [5,6,7]. Backfill mining as a green mining method can reduce surface damage and effectively dispose of solid waste such as CG [8]. CG has poor cementation properties and is mainly used as aggregate in backfill mining [9,10]. However, the high carbon content of CG leads to poor interfacial bonding of the filler, which affects the performance of the cemented paste backfill (CPB) [11,12]. Borrowing the technical idea of metal mine tailings activation for clinker preparation [13,14,15], decarbonization and activation treatment of CG can fully use CG’s heat and stimulate its activity, which is essential to realize the resourceization and sustainable utilization of CG.

Thermal activation is considered to be the most promising method for destroying the crystal structure of CG and improving its reaction activity [16]. Much research has been carried out to find the best activation process to achieve high chemical reactivity of CG. Organic components and carbon in CG can be burned off after calcination, and kaolinite in CG can also be gradually converted to meta kaolinite in the temperature range of 500–800 °C, thus improving the activity of volcanic ash [17,18,19]. Hao [20] investigated the effect of thermal activation conditions on the mineral phase and structural changes of CG minerals from the Junggar coalfield, Inner Mongolia, China, and showed that the highest volcanic ash activity was reached when calcined at 800 °C for 2 h. Song [21,22] calcined the Xuzhou CG and the results showed that the optimum thermal activation process for CG was 700 °C kept warm for 2 h when it showed the highest reactivity. Frías et al. [23] calcined 5 different samples of CG at different temperatures (500–900 °C) with a holding time of 2 h and used them in cement mortar to replace 12% of cement calcined at 800 °C. The cement mortar with coal CG had 18% higher compressive strength at 28 days than the control group. The results show that the volcanic ash produced by calcination at 600~800 °C has the best activity. However, the required calcination time is 1–2 h, and the long time required for thermal activation seriously restricts the scale of CG thermal activation.

Preheated combustion is an effective way to achieve short-time combustion of low-volatile fuels [24]. A new technology of preheated combustion of pulverized coal was first proposed by the All-Russian Institute of Thermal Engineering [25], where pulverized coal was preheated to about 816 °C and then burned with a minimum fluidization velocity of 0.4 m/s, which ensured pulverized coal burnout. The Institute of Engineering Thermophysics, Chinese Academy of Sciences, proposed a circulating fluidized bed preheating coupled with pulverized coal furnace combustion technology [26,27,28,29], where the circulating fluidized bed ignites and preheats the fuel in the first stage, and stable and efficient combustion of low-volatile-content fuel can be achieved in the pulverized coal furnace in the second stage with a minimum fluidization velocity of 0.67 m/s. Lyu and Wang Shuai et al. [30,31] used a two-stage descending tube furnace to achieve preheated combustion, studied the effects of preheating temperature, residence time, and combustion temperature on combustion exhaustion, and concluded that the pulverized coal could be fully combusted at a preheating time of 0.4 s. Wang Xuebin [32] developed the technology of constant temperature preheat decarburization of low-volatile-matter and low-calorific-value fuels. The 1st generation plant has been in stable operation for 2 years in Ganquanbao, Xinjiang, with an annual treatment of 600,000 tons of gasification slag, a combustion temperature of 900 °C, and a combustion time of 3.7–5.6 min. It has been proved that the preheat combustion technology can burn out the residual carbon of low-volatile-matter fuels in a short time. However, there are few studies on preheat decarbonization of CG, the activity of the CG residue after preheating decarbonization is unknown, and relevant research on the preparation of paste filling material as a gelling material has not been carried out. Therefore, preheat decarbonization activation of coal CG and carrying out preheat decarbonization CG-based CPB material research to promote the resource utilization of CG residue carbon to achieve sustainable consumption of CG activation scale has essential significance.

In order to fill the relevant gap in the literature, this paper prepares a cemented paste backfill with preheated decarburized activated CG (PCG), FA, activator, low-dose cement (most backfill materials use more than 10% cement dosage [33,34,35,36]), and water based on short-time preheated decarburization activation of CG. The innovations of this study are mainly in three aspects: (1) to propose a new idea of CG decarbonization activation and a new PCCPB using the described method; (2) to research the effect law of various Na_2_SO_4_ dosages on the performance of novel PCCPB; and (3) to reveal the micromechanism of novel PCCPB by means of FTIR, TG/DTG, XRD, and SEM. The research content provides new methods for decarbonizing and activating CG and large-scale utilization, promoting green and sustainable coal mining.

## 2. Materials and Methods

### 2.1. Materials

The raw materials for this experiment are PCG, fly ash (FA), aeolian sand (AS), cement (OPC), activator, and water. PCG and FA served as auxiliary materials, cement as cementitious materials, AS as aggregate, municipal water as mixing water, and Na_2_SO_4_ as an activator.

#### 2.1.1. Preheated Decarburized Activated CG

**Figure 1:**
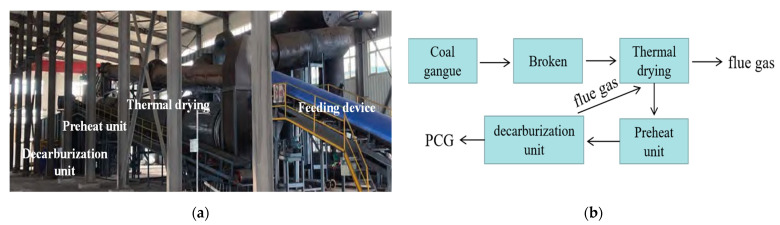
Equipment preparation process. (**a**) Site operation drawing of equipment; (**b**) flow chart of preheating decarburization.

**Figure 2:**
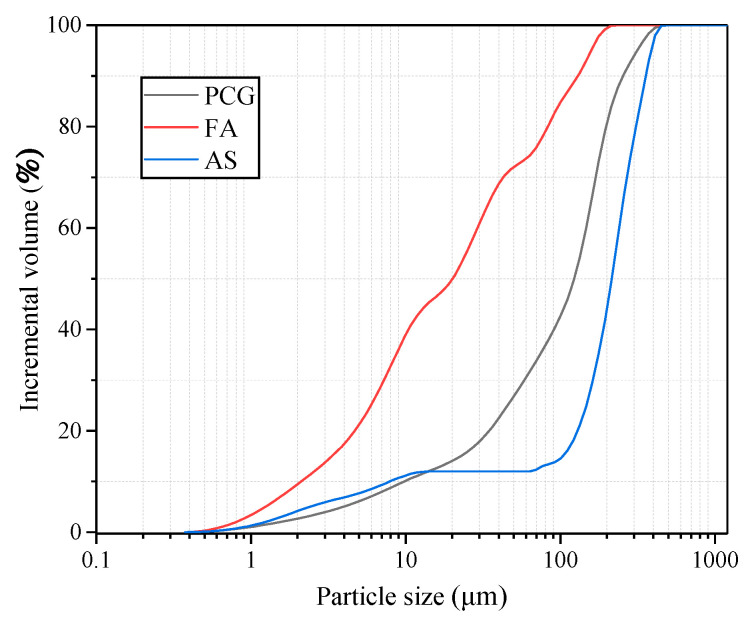
Particle size distribution of PCG, FA, and AS.

**Figure 3:**
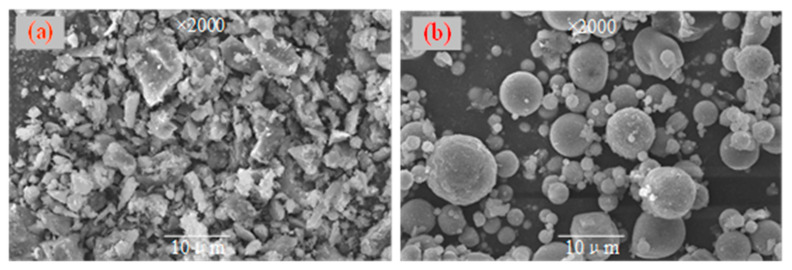
SEM result. (**a**) PCG; (**b**) FA.

**Figure 4:**
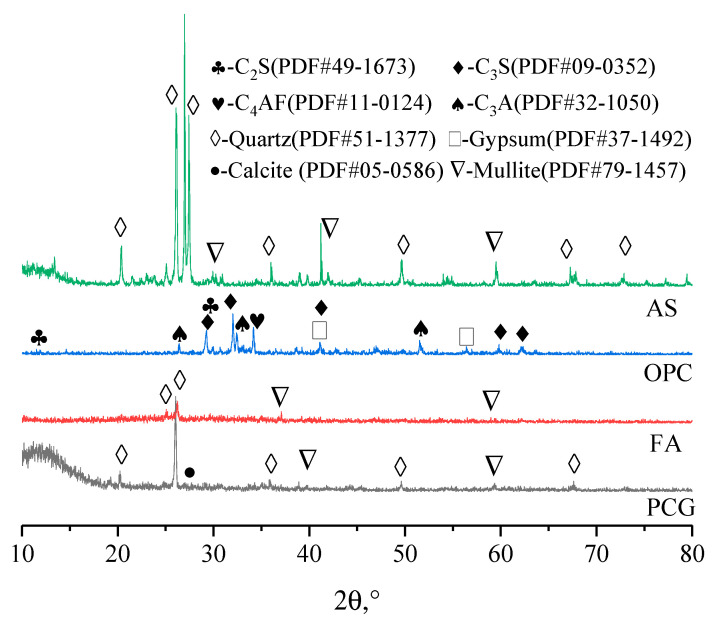
XRD result of raw materials.

The PCG used in the experiment was prepared using a preheated decarburization 108 device in Ganquanbao, Xinjiang, China. Its preparation process is shown in Figure 1. The device is based on the thermostatic preheated decarburization technology developed by Wang Xuebin [32], and a CG combustion temperature of 900 °C and combustion time of 4.0 min were used in this experiment. The PCG was ground for 10 min in the laboratory using a ball mill (SM-500, Wuxi, China) as this experimental material. Next, PCG’s particle size distribution (PSD) after grinding was detected based on a laser scanner (Malvern 2000; Malvern, UK), as shown in Figure 2. The PSD curves indicated that the experimental coarse particles had relatively low PCG content. Moreover, the chemical properties of the prepared PCG were assessed, and the PCG was tested using an X-ray fluorescence spectrometer (XRF) (ARL spectrometer, Waltham, MA, USA). The chemical composition of PCG was tested (Table 1) and the main chemical constituents were SiO_2_ (53.55%), Al_2_O_3_ (20.41%), and Fe_2_O_3_ (8.99%). In addition, PCG’s microscopic morphology and mineral composition were tested by XRD (D8 Advance, Karlsruhe, Germany) and SEM (JSM-7610F, Akishima-shi, Japan). A SEM image of PCG with 2000 times magnification is shown in Figure 3, which indicates that the microscopic morphology of PCG is irregular with an angular shape. The mineralogical composition of PCG is shown in Figure 4. PCG is mainly composed of quartz and mullite. Moreover, the specific surface area of PCG was tested based on a specific surface area meter (BSD-660, Beijing, China) with the result of 3.17 m^2^/g.

#### 2.1.2. Fly Ash

The FA was bought at the pineapple power plant in Yulin, Shaanxi Province, and the same analytical method as PCG was used for FA. Figure 2 and Table 1 show the *d_10_*, *d_60,_* and *d_90_* values of FA as 2.24 μm, 33.0 μm, and 135.3 μm, respectively. The PSD curves show that the content of the fine particles is shallow. The results of FA constituent testing are exhibited in Table 1. The main components are Al_2_O_3_, SiO_2_, and Fe_2_O_3_ accounting for 86.65%. The microscopic morphology of FA is small and granular. The XRD detecting results are shown in Figure 4. The main minerals of fly ash are aluminosilicate (50–85%), sponge-like vitreous (10–30%), quartz (1–10%), iron oxide (3–25%), carbon particles (1–20%), and sulfate (1–4%).

#### 2.1.3. Aeolian Sand

The AS used in the experiments was taken from Yuyang District, Yulin City. The main light minerals of AS are quartz, feldspar, and calcite, accounting for more than 90%. Heavy minerals are amphibole. Mica and epidote account for more than 5%. Figure 2 and Table 1 show the *d_10_*, *d_60,_* and *d_90_* values of AS as 8.27 μm, 256.03 μm, and 357.69 μm, respectively. The PSD curves show that the experiment’s acceptable particle content is shallow, and the corresponding gradation is relatively discontinuous. The composition test results of AS can be seen in Table 1. The main components are SiO_2_ and Al_2_O_3_, accounting for 78.1%.

#### 2.1.4. Cement, Activator, and Water

Cement is a cementitious material, and its main mineralogical components are C_2_S (20%), C_3_S (50%), C_3_A (7–15%), C_4_AF (10–18%), etc. The main chemical composition is shown in Table 1. The main hydration products are calcium hydroxide (CH), calcium silicate hydrate (C-S-H), calcium aluminate hydrate (C-A-H), and calcium alumino-ferrite hydrate (C-A-F-H). The cement type used is ordinary Portland cement (OPC) 42.5, and the basicity of the cement is not more than 0.6%, in line with the Chinese national standard GB175-2007. Na_2_SO_4_ was selected as the activator. It was produced based on the standard of GB/T 9853-2008. The mixing water was mixed with municipal tap water.

### 2.2. Sample Preparation

According to the preliminary experimental study, it was determined that the PCCPB solid mass concentration of this experiment was 78%, the cement dosage was 3% of the total solid mass, the ash–sand ratio was fixed at 0.5, and the PCG, FA, cement, AS, and activator were formulated as shown in Table 2. The numbers in the table indicate different levels.

### 2.3. Experimental Setup and Method

#### 2.3.1. Mechanical Property Test

Under the condition that PCCPB samples reached curing time (3 d, 7 d, 14 d, and 28 d), the UCS tests were conducted with a condition of 1.0 mm/min based on a DNS100 system (SinoTest, Changchun, China) in terms of the standard of GB/T 50081-2019. All experiments were performed three times, and the mean value of UCS was taken.

#### 2.3.2. Microstructure Test

Fourier-transform infrared spectroscopy (FTIR) tests were performed on PCCPB samples that reached the curing time (3 d, 7 d, 14 d, and 28 d). FTIR tests were performed using a Nicolet iN10 Fourier transform micro-infrared spectrometer (Thermo Fisher, Waltham, MA, USA).

The change in weight loss with temperature was tested for cured 3 d and 28 d PCCPB samples using a TGA5500 device. Nitrogen was applied to block carbonization during the heating process. The heating rate in this experiment was 20 °C/min to 900 °C.

The Bruker X-ray diffractometer was used to test the PCCPB samples with a curing time of 3 d and 28 d. Then, the mineral composition of the samples was determined by comparing JADE6.0 software with powder diffraction file (PDF) cards. After the completion of the UCS test, a sample from the central part of the sample was selected and soaked for 48 h to terminate the hydration process. A scan rate of 5°/min and a spectral range of 5° to 80° were used for the tests.

The microstructure of the curing times of 3 d and 28 d specimens was observed using a JEOL JSM-6460LV SEM device. After the UCS test, the central part of the sample was selected and cut into 2 mm slices, which were put on the sample stage for testing.

#### 2.3.3. Leaching Toxicity Test

**Figure 5:**
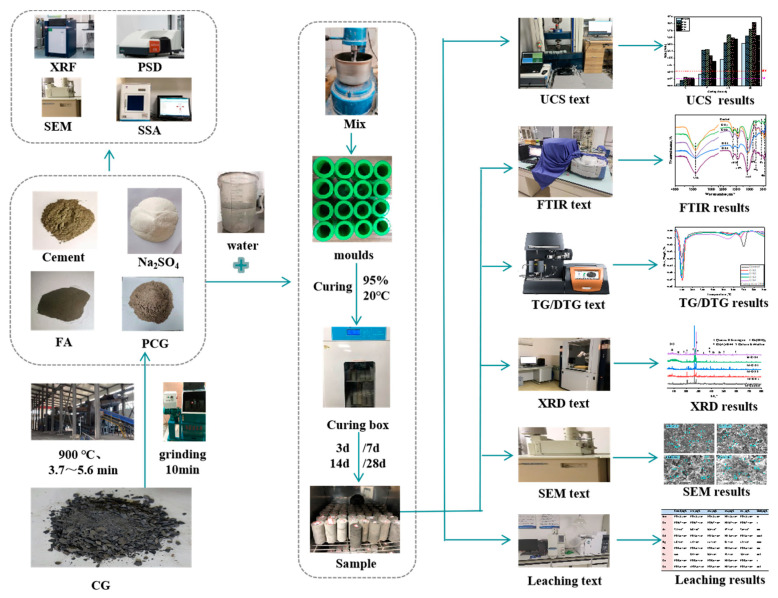
Experimental flowchart.

The samples were crushed so that all sample particles passed a 3 mm sieve. The “Leaching Toxicity Leaching Method for Solid Wastes Inverted Shaking Method” (HJ557-2010) [37], using pure water as a leaching agent, was used. This standard assesses the likelihood that inorganic contaminants would leach from solid wastes and other solid materials into the surface or groundwater [38]. Figure 5 illustrates the test procedure.

## 3. Results and Discussion

### 3.1. Mechanical Performance of PCCPB

#### 3.1.1. Analysis of UCS of PCCPB

The mechanical properties of the PCCPB are critical indicators to evaluate its stability, which can be expressed by its UCS, coefficient of variance (CV) of UCS, and strong growth rate. Table 3 shows the results of the fundamental statistical analysis of the UCS of PCCPB, and the results demonstrate the mean ($\bar{X}$), standard deviation (SD), and coefficient of variance (CV). SD and CV measure the degree of dispersion of the data, where CV can be expressed by Equation (1) [39]. The SD of all PCCPB samples was kept below 0.13, and the CV was below 15% [40], indicating that the test data are available.

(1) $$\mathrm{CV}=\frac{\mathrm{SD}}{\bar{X}}\times 100\%\;$$

**Figure 6:**
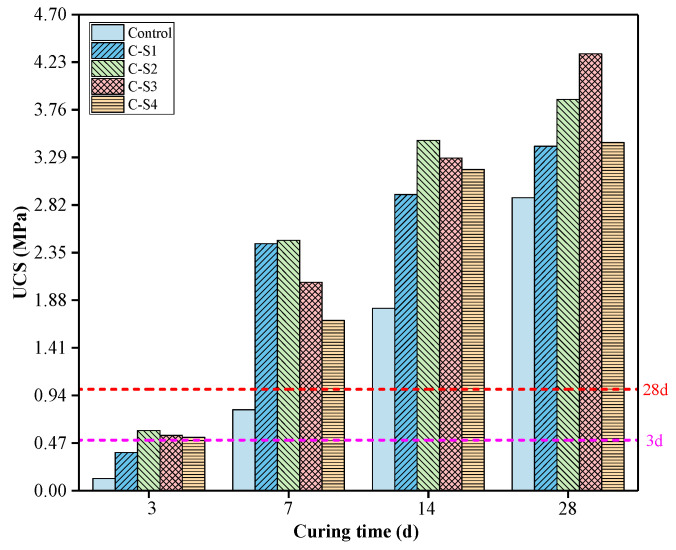
Effect of Na_2_SO_4_ dosage on the UCS of PCCPB.

The variation of the UCS of PCCPB samples with the curing time and Na_2_SO_4_ dosage is exhibited in Figure 6. The UCS of PCCPB samples is positively related to curing time, which has been shown by other studies [41,42]. As can be seen from Figure 6, the UCS of C-S3 enhanced from 0.545 MPa (3 d) to 2.057 MPa (7 d), 3.284 MPa (14 d), and 4.312 MPa (28 d) after curing 3 d, 7 d, 14 d, and 28 d, respectively. The increase in UCS is caused by a large number of hydration products (C-(A)-S-H, calcium alumina, CH, and silicate, etc.) filling the internal pores, which leads to the higher compressive strength of the material, a conclusion confirmed in Section 3.2.4, the research of Liu et al. also confirmed this conclusion [43,44].

From Figure 6, it can be seen that Na_2_SO_4_ has an excitation effect on PCCPB samples at different curing times, and the UCS of PCCPB increases and subsequently decreases during the process of increasing Na_2_SO_4_ dosage. Comparing the UCS of PCCPB at 3 d, 7 d, and 14 d, the highest UCS was found in the C-S2 group, indicating that adding 2% Na_2_SO_4_ had an apparent enhancement influence on the early strength of PCCPB [45], which indicated that more Na_2_SO_4_ in the early reaction process would inhibit the hydration reaction. For the curing time of PCCPB for 28 d, the UCS of C-S1, C-S2, C-S3, and C-S4 enhanced by 17.61%, 33.62%, 49.15%, and 18.89%, respectively, compared with the control group (the rates of increase in UCS of other groups are listed in the following table), and the UCS corresponding to the C-S3 group was the highest. When the addition of Na_2_SO_4_ dosage exceeded 3%, its UCS showed a decreasing trend, probably because the Na_2_SO_4_ that did not participate in the reaction remained in the pores of the PCCPB samples and produced sulfate erosion of the hydration products later [46].

According to the mechanical requirements of the CPB (3 d ≥ 0.5 MPa and 28 d ≥ 1.0 MPa) [47,48], the mechanical properties of C-S2, C-S3, and C-S4 meet the mechanical requirements of the CPB, and the UCS data of 3 d and 28 d were combined to determine CS3 as the best ratio.

#### 3.1.2. Analysis of Elastic Modulus of PCCPB

The elastic modulus (EM) is a physical and mechanical parameter that directly affects the performance of the PCCPB and is also a necessary parameter for stability analysis, optimization of the structural parameters of the quarry, and numerical simulation. The EM can reflect the degree of bonding between aggregate particles in the PCCPB, and increasing the EM of the PCCPB can reduce the damage through the elastic buffering effect, thus improving the stability of the PCCPB [49].

**Figure 7:**
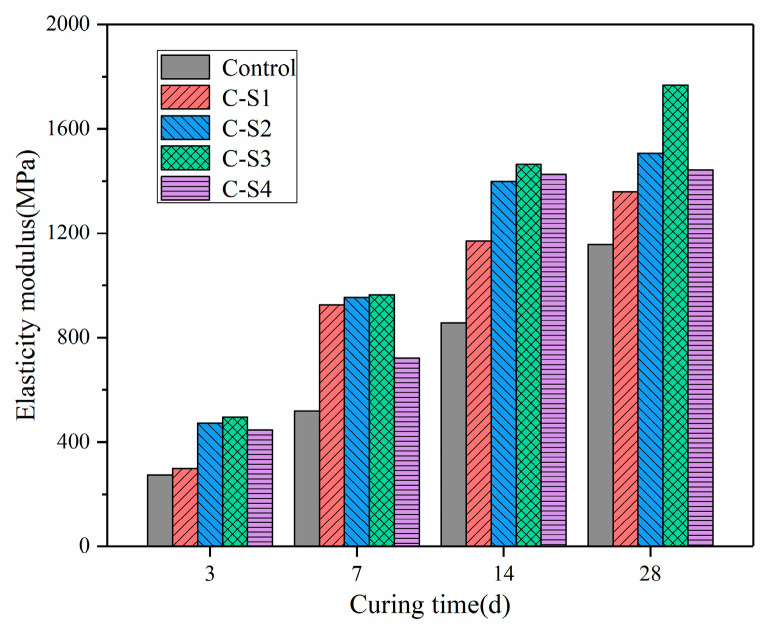
The EM of PCCPB with different Na_2_SO_4_ dosages.

Figure 7 shows the EM of PCCPB with different Na_2_SO_4_ dosages. The effect of Na_2_SO_4_ on the EM of PCCPB is similar to that of UCS, where the EM at any curing time increases with Na_2_SO_4_ and then decreases, with the highest EM corresponding to the C-S3 group and the most vigorous resistance to deformation of the PCCPB [50]. The EM of the C-S3 group increased by 80.1%, 85.9%, 29.5%, and 52.9% at 3 d, 7 d, 14 d, and 28 d of curing time, respectively, compared with the control group. Under the addition of Na_2_SO_4_ of more than 3%, the EM showed a decreasing trend.

**Figure 8:**
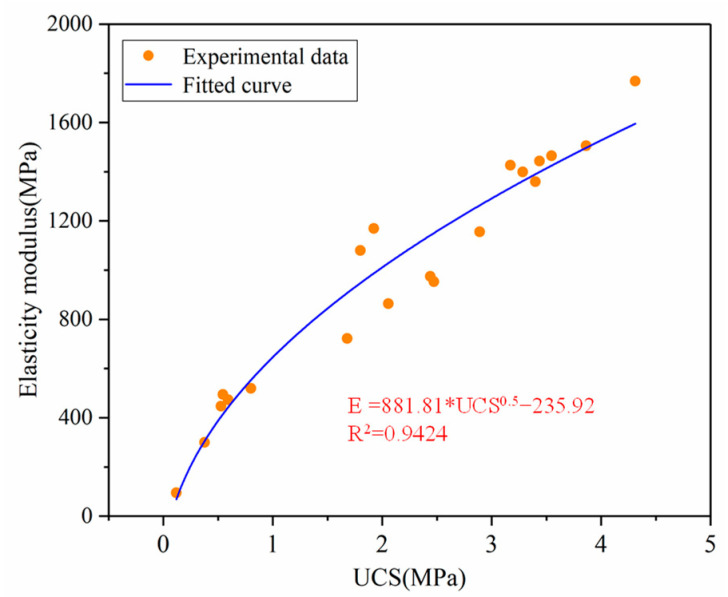
Fitting relationship between elastic modulus and UCS of PCCPB.

Figure 8 reflects the variation of the EM of PCCPB with UCS, which shows that as the UCS increases, the EM increases, and vice versa. The relationship between UCS and EM of PCCPB was analyzed by the regression analysis method, including the square root of compressive strength, cube root of compressive strength, and quadratic function of UCS. The square root of UCS was found to be more suitable to describe the relationship between UCS and EM, and red mud material showed a similar relationship [51]. The final equation derived from the analysis is shown in Equation (2):

(2) $$E=A\times {\mathrm{UCS}}^{0.5}+B$$

where E represents the EM (MPa), and A and B are fitting parameters.

The fit coefficient *R*^2^ = 0.9424 indicates that a quadratic function using the UCS’s square root is consistent with the inspired relationship between the EM and the UCS of the PCCPB.

### 3.2. Microstructure of ARFGB

#### 3.2.1. FTIR Results Analysis of PCCPB

**Figure 9:**
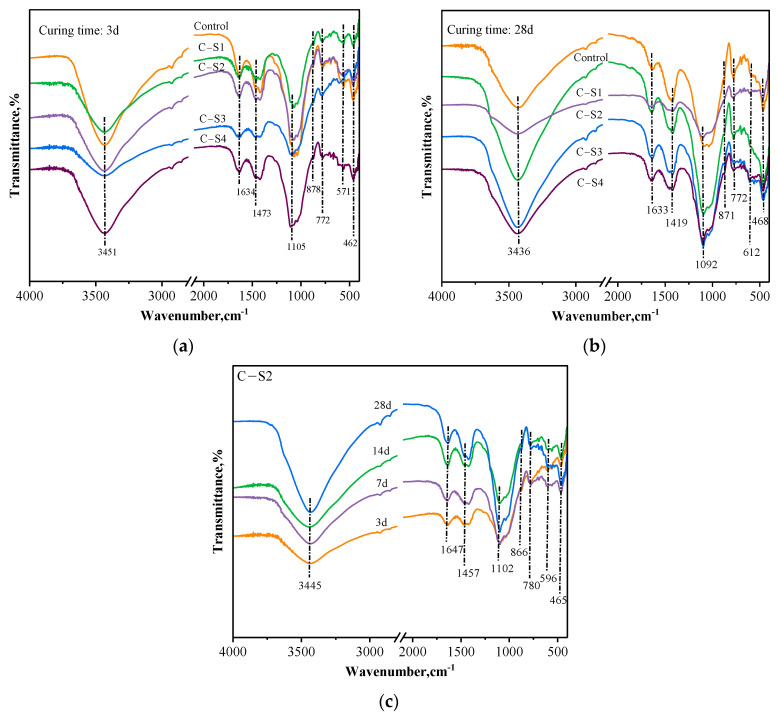
FTIR results of PCCPB samples. (**a**) 3 d; (**b**) 28 d; (**c**) C-S2.

The Fourier-transform infrared spectra (FTIR) of the PCCPB samples are shown in Figure 9. The bands corresponding to different Na_2_SO_4_ dosages and different curing times of the PCCPB samples shown in Figure 9 are around 3436 cm^−1^, 1633 cm^−1^, 1419 cm^−1^, 1092 cm^−1^, 871 cm^−1^, 772 cm^−1^, 612 cm^−1^, and 463 cm^−1^, respectively. The absorption peaks near 3436 cm^−1^ and 1633 cm^−1^ are O-H stretching vibration peaks and bending vibration peaks, respectively. These O-H characteristic absorption peaks originate from the structural water produced by the hydration reaction and a small part of the free water in the system [52]. The absorption peak near 468 cm^−1^ is due to the bending vibration of the Si-O bond [53]. This band represents quartz. The absorption peaks near 612 cm^−1^ are bending vibrations of Al-O, which correspond to the [Si(OH)_5_]^−^ monomeric structures in the 3D mesh of the gelling material [54]. The absorption peak near 871 cm^−1^ corresponds to the Si-OH band’s bending vibration [55]. The band at 1419 cm^−1^ indicates that O-C-O bonds stretch in the presence of carbonates associated with calcite in the sample [56]. The peak near 772 cm^−1^ corresponds to the symmetric stretching vibration of Si-O-T dissolved in SiO_4_ tetrahedra; thus, the dissolved Si-Al elements are involved in forming C-(A)-S-H gels; this is also confirmed by Li et al. [17]. The peak near 1092 cm^−1^ is the stretching vibration peak of asymmetric Si-O-Si or Si-O-Al. These absorption peaks show a structure of the Si-O-Al-O bond interconnection in the system. The [Si(OH)_5_]^−^ and [Al(OH)_4_]^−^ monomer structures in the system are connected by these bonds to form a polymer, which recombines into a three-dimensional network structure of the cementitious material [57].

#### 3.2.2. TG-DTG Results Analysis of PCCPB

**Figure 10:**
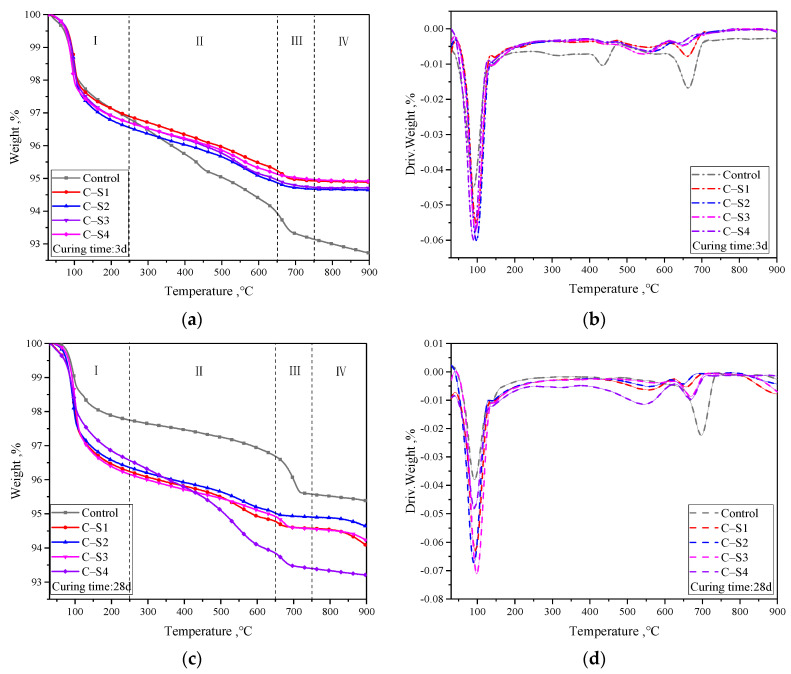
TG-DTG results of PCCPB samples. (**a**) 3d sample mass and temperature relationship (**b**) 3d sample mass loss rate of change and temperature (**c**) 28d sample mass and temperature relationship (**d**) 28d sample mass loss rate of change and temperature Table 4 reflects the weight loss rates of PCCPB with various Na_2_SO_4_ dosages, and the magnitude of the weight loss rate indirectly reflects the number of hydration products in a given temperature interval [62]. In the I zone, the weight loss of free water, C-(A)-S-H, and AFt in PCCPB was the first to increase and then to decrease with the increase in Na_2_SO_4_ dosage. The most significant weight loss in the PCCPB samples for curing time of 3 d was C-S2, which was 3.45% (zone I), of which the free water weight loss represented a relatively large ratio. The most significant weight loss in the PCCPB samples for a curing time of 28 d was C-S3, which was 3.84% (zone I), with C-(A)-S-H and AFt accounting for a relatively large proportion of the weight loss. This is consistent with the findings in Section 3.1 and Section 3.2.4. In addition, mass loss in zone I was found to be higher for all samples at 28 d than at 3 d, indicating that PCCPB continued hydration with increasing age. This is consistent with the studies of Zhang et al. [63] and Feng et al. [64].

Figure 10 exhibits the TG–DTG data for different Na_2_SO_4_ dosages and curing times of PCCPB samples. By and large, all TG–DTG curves have similar characteristics with three prominent heat absorption peaks in the temperature ranges of 50–250 °C (zone I) and 400–450 °C (II). The heat absorption peak at 50–250 °C is associated with the evaporation of free water and dehydration of hydration products (C-(A)-S-H and AFt) [58]. The heat absorption peak at 400–450 °C is assigned to CH dehydration, and the decomposition of calcium carbonate and CH occurs at 600–750 °C [59]. In addition, a weaker heat absorption peak in zone IV above 750 °C is associated with the dehydroxylation of silicate minerals in the sample [60,61]; the weight loss reactions in zones I, II, and III also reveal the hydration reaction products of PCCPB, and the results are consistent with Section 3.2.3.

#### 3.2.3. XRD Results Analysis of PCCPB

**Figure 11:**
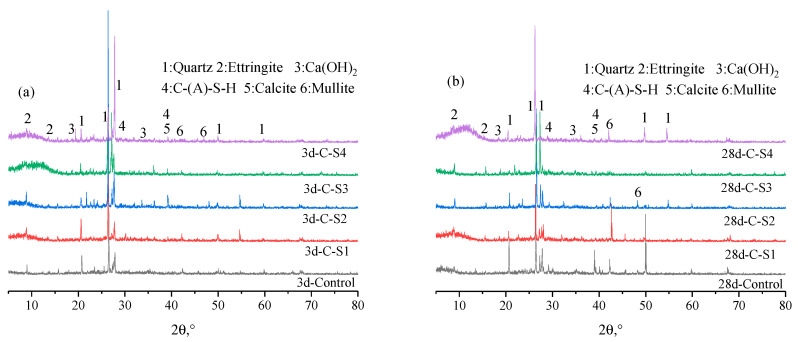

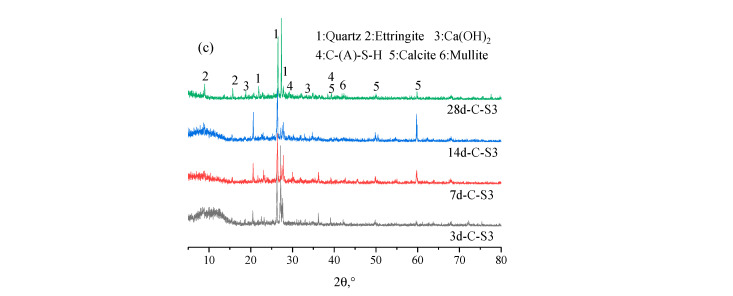
XRD results of PCCPB. (**a**) 3 d; (**b**) 28 d; (**c**) C-S3.

Figure 11 shows the XRD results of PCCPB. The diffraction peaks of quartz (PDF # 51–1377), ettringite (PDF # 37–1476), calcium hydroxide (PDF # 84–1263), C-(A)-S-H gel (PDF # 34–0002), calcite (PDF # 41–1475), and mullite (PDF # 83–1881) are observed. This is consistent with the results of Mota et al.’s [65] study on cement hydration products in pure cement and sodium sulfate environments. Calcium alumina, calcium hydroxide, and C-(A)-S-H gel are the hydration products generated in the specimens. The primary source of both is from both generation channels. The hydration of cement generates 1, see Equation (3)—the reaction of reactive SiO_2_ generates 4 [66,67] and the other and Al_2_O_3_ in FA and PCG with CH in the hydration reaction of silicate cement, see Equations (5)–(7) [68,69]. The second channel, calcium alumina, is formed by the reaction of Al_2_O_3_ with CH to form C-A-H and then with gypsum. The formula for C-A-S-H is given in Equation (8). According to the XRD pattern of the original material, it contains quartz and mullite minerals and does not participate in the hydration reaction [70]. The calcite is derived from both the raw material and the specimen. Calcite is derived from the specimen’s raw material and surface charring.

(3) $$3\mathrm{CaO}\cdot {\mathrm{Al}}_{2}O_{3}{(C}_{3}{A)+3\mathrm{CaSO}}_{4}\cdot {2H}_{2}{O+30H}_{2}O\to \mathrm{CaO}\cdot {\mathrm{Al}}_{2}O_{3}\cdot {3\mathrm{CaSO}}_{4}\cdot {32H}_{2}O(\mathrm{AFt})$$

(4) $$3\mathrm{Ca}\cdot {\mathrm{SiO}}_{2}{+\mathrm{xH}}_{2}O\to \mathrm{xCaO}\cdot {\mathrm{SiO}}_{2}\cdot {\mathrm{yH}}_{2}{O(C-S-H)+(3-x)\mathrm{Ca}(\mathrm{OH})}_{2}$$

(5) $${\mathrm{Al}}_{2}O_{3}{+\mathrm{Ca}(\mathrm{OH})}_{2}{+\mathrm{xH}}_{2}O\to \mathrm{CaO}\cdot {\mathrm{Al}}_{2}O_{3}\cdot {\mathrm{xH}}_{2}O(C-A-H)$$

(6) $$3\mathrm{CaO}\cdot {\mathrm{Al}}_{2}O_{3}\cdot {6H}_{2}{O+3\mathrm{CaSO}}_{4}{+26H}_{2}O\to 3\mathrm{CaO}\cdot {\mathrm{Al}}_{2}O_{3}\cdot {3\mathrm{CaSO}}_{4}\cdot {32H}_{2}O(\mathrm{AFt})$$

(7) $${\mathrm{SiO}}_{2}{+\mathrm{Ca}(\mathrm{OH})}_{2}{+\mathrm{xH}}_{2}O\to \mathrm{CaO}\cdot {\mathrm{SiO}}_{2}\cdot {\mathrm{xH}}_{2}O(C-S-H)$$

(8) $${\mathrm{Al}}_{2}O_{3}{+\mathrm{Ca}(\mathrm{OH})}_{2}{+2\mathrm{SiO}}_{2}{+\mathrm{xH}}_{2}O\to \mathrm{CaO}\cdot {\mathrm{Al}}_{2}O_{3}\cdot {2\mathrm{SiO}}_{2}\cdot {\mathrm{xH}}_{2}O(C-A-S-H)$$

From the result in Figure 11a, quartz, calcite, and mullite diffraction peaks do not change significantly in all plots as the Na_2_SO_4_ dosage increases from 0 wt.% to 4 wt.%. The variation is more significant for calcium alumina and C-(A)-S-H gels, where the intensity of the diffraction peaks of these two hydration products first becomes stronger from a weak diffraction peak and then changes to a weak diffraction peak. Specifically, the dosage of Na_2_SO_4_ increased from 0 wt.% to 2 wt.%, and the diffraction peak of chalcocite at 2θ was enhanced at 9°. However, the dosage of the Na_2_SO_4_ was increased to 4 wt.%, and the diffraction peak of chalcocite in the C-S4 sample was decreased. In addition, a similar phenomenon was observed for the C-(A)-S-H gel diffraction peak at 2θ of 39°. This implies that the diffraction peaks of the hydration products in the C-S2 specimens are the strongest at the curing time of 3 d. This is consistent with the UCS of all specimens at 3 d. This suggests that Na_2_SO_4_ as an activator facilitates the generation of calcium alumina and improves early strength development, which agrees with Razali et al. [71] and Zhao et al. [72]. However, with excess Na_2_SO_4_, the slurry viscosity increases, leading to the precipitation of C-(A)-S-H gel in the early stage, which inhibits the positive proceeding of hydration [73,74]. In addition, comparing Figure 11a,b, it can be seen that the diffraction peaks of calcite and C-(A)-S-H gels in the curing time of 28 d of PCCPB were significantly enhanced, especially for calcite with 2θ of 16°. The diffraction peaks of calcite with 2θ of 49° were significantly enhanced compared with those in the 3 d samples, especially for the C-S4 sample. Notably, the diffraction peaks of calcium hydroxide with 2θ of 18° and 34° do not change significantly in all samples.

On the one hand, it may be due to the small cement content used in this case of only 3 wt.% of the solid mass, which generates less calcium hydroxide. On the other hand, it may be that the excitation of Na_2_SO_4_ promotes the hydrolysis of calcium oxide in FA, replenishing the calcium hydroxide consumed by hydration. In order to analyze the effect of curing time on the hydration products, C-S3 samples were selected for testing, and the curing time of the specimens was 3 d, 7 d, 14 d, and 28 d. The XRD results are shown in Figure 11c. The diffraction peaks of the hydration products (calcium alumina, C-(A)-S-H gel) in the samples changed from weak to firm as the curing time increased from 3 d to 28 d. This indicates that the content of hydration products increases with the curing time, increasing strength. Ouyang et al. [75] also obtained a similar conclusion and found that the diffraction peaks of the hydration products (AFt, calcium alumina, and C-(A)-S-H gel) of the sand-based cemented paste filling material were enhanced with the extension of curing age from 1 d to 7 d and 28 d.

#### 3.2.4. SEM Results Analysis of PCCPB

**Figure 12:**
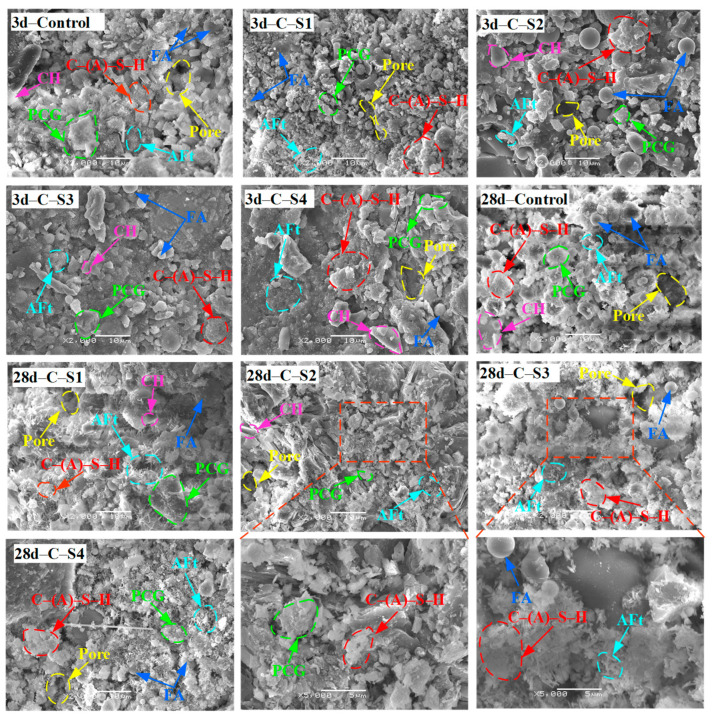
SEM results of PCCPB.

Figure 12 shows the SEM results of PCCPB samples for the curing times of 3 d and 28 d. It was observed that the microscopic morphology of PCCPB samples showed significant differences depending on curing time and the ratio of Na_2_SO_4_. With the increase in Na_2_SO_4_, the microscopic morphology of all PCCPB samples showed a pattern from loose to dense and slightly loose. Under the condition of a curing time of 3 d, the control group was the loosest and most porous. When Na_2_SO_4_ was increased to 2 wt.%, the microscopic morphology of the C-S2 group changed more, from loose to relatively dense, and the matrix of small granular FA and PCG was reduced. After curing for 28 d, abundant needle-like calcarenite (whose length is greater than 1 μm) and irregularly shaped C-(A)-S-H gels were observed in microscopic images of all samples, which had relatively more hydration products and a more extensive distribution range compared with the 3 d samples. The intercalation of calcarenite between the pores of PCCPB increases the denseness of the specimens. However, the control group has fewer hydration products and a relatively loose structure. The microstructure of PCCPB is mainly composed of C-(A)-S-H gels, fine needle-like AFt crystals, hexagonal lamellar CH, unhydrated AS particles, and PCG. This differs from the hydration products of binders made from blast furnace slag [76,77], and the reported results for the hydration product of pure cement are consistent [78,79]. In addition, the formation of hydration products provides the basis for the UCS of C-(A)-S-H.

**Figure 13:**
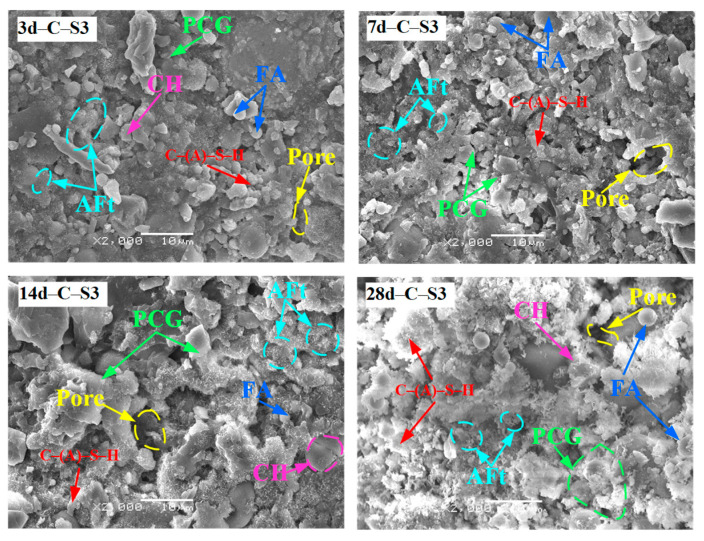
SEM results of C-S3 group samples.

Figure 13 shows the SEM results of C-S3 group samples. From Figure 13, it can be seen that the microstructure of the samples becomes dense as the age increases from 3 d to 28 d with the gradual increase in calcium alumina (AFt) and C-(A)-S-H gel. It was also found that the number of unreacted FA and PCG particles decreased. This is because the silica–oxygen tetrahedral monomer and aluminum–oxygen tetrahedral monomer in FA and PCG are involved in the generation of C-(A)-S-H gels and are connected, gradually forming a monolithic structure [80]. The hydrated calcium silica-aluminate structure has a specific skeletal effect [81,82], thus increasing the UCS of the samples with curing time. This is consistent with the findings of the previous UCS and XRD studies. Zhang and Tang et al. [55] reached similar conclusions. For example, Tang et al. [83] studied fly ash–aeolian sand filling materials and found that AFt and C-(A)-S-H gels in the samples increased with the extension of curing age, and the micro-morphology of the samples changed from loose to dense.

### 3.3. Leaching Toxicity Results Analysis of PCCPB

When using PCCPB as a backfill material, it is necessary to evaluate the impact of PCCPB weight metal ion leaching on groundwater [84]. Table 5 exhibits the leaching data of heavy metal ions from PCCPB in 28 days of curing. The standard limits are taken from HJ/T 300-2007, which show that the heavy metal ions of leachate in the control group and CS1-CS4 group meet the requirements of groundwater class III standard, which indicates that PCCPB meets the environmental safety requirements and PCCPB is a promising technology for heavy metal curing and stabilization. The CS1-CS4 group is lower than the control group, and the CS3 group is the lowest for three main reasons: (1) C-(A)-S-H and CH are formed by PCCPB hydration, as shown in Section 3.2.2 and Section 3.2.3. C-(A)-S-H and CH can immobilize heavy metals on the gelling particles through chemical bonding and physical adsorption/wrapping microstructure [79,85,86]. The lowest CS-4 value is attributed more to C-(A)-S-H and CH products (Section 3.2.3), confirmed by its macroscopic uniaxial compressive strength. (2) Calcareous aluminate neutralizes heavy metal ions within the needles by chemical replacement. There is ample evidence that calcarenite can trap heavy metal elements such as Cd and Cu [87]. (3) The solidification/stabilization of Cr by PCCPB is due to the physical adsorption of hydration products such as C-(A)-S-H gels and sodalite. In addition, hydration refines the pore structure and blocks the transport channels of Cr ions, facilitating solidification/stabilization [88]. Calcite, calcium silicate, calcium hydroxide, and calcium silicate may play a key role in immobilizing As species and heavy metals in PCCPB [89].

## 4. Conclusions

This study comprehensively evaluated the properties of a novel PCCPB prepared from PCG, FA, activator, low-dose cement, and water. The effects of different activator dosages on the mechanical properties, microstructure, and leaching risk of PCCPB were investigated. Based on the experimental results, the following main conclusions can be drawn:

(1) The UCS of C-S2, C-S3, and C-S4 can meet the requirements of backfill mining, among which the UCS of C-S3 is 0.545 and 4.312 MPa at 3 d and 28 d, respectively. Na_2_SO_4_ has an excitation effect on PCCPB at different curing times, and the UCS of PCCPB increases and then decreases with the increase in Na_2_SO_4_. The 3% Na_2_SO_4_ has the best excitation effect on the later strength (28 d) of PCCPB.
(2) The main hydration products of PCCPB are C-(A)-S-H gel and calcium alumina (AFt), and the effect of different Na_2_SO_4_ dosages on the content and micromorphology of the gelling products of PCCPB is the main reason for changing its mechanical properties and leaching risk.
(3) All groups’ (control group and CS1–CS4 group) leachate heavy metal ions meet the groundwater class III standard requirements, and PCCPB meets the environmental safety requirements. Based on the mechanical properties and leaching results, C-S3 was the best ratio.
(4) Preheated decarburization (combustion temperature 900 °C, time 4 min) is an effective method of CG activation, and with PCG, FA, activator, low-dose cement, and water, preparation of the novel PCCPB is feasible.

## Figures and Tables

**Table 1:** Main chemical compositions of PCG, FA, AS, and OPC.

Chemical Composition	OPC	AS	PCG	FA
Al_2_O_3_	5.53%	10.30%	20.41%	17.14%
SiO_2_	22.36%	67.80%	53.55%	41.01%
CaO	65.08%	5.30%	3.76%	14.03%
Fe_2_O_3_	3.46%	5.80%	8.99%	14.47%
K_2_O	0.62%	7.50%	1.62%	5.36%
Mg_2_O	1.27%	1.78%	3.65%	4.31%
TiO_2_	0.59%	0.35%	0.73%	1.60%
SO_3_	/	/	1.27%	0.711%
Others	1.09%	1.17%	5.62%	0.83%

**Table 2:** Mixing proportions of PCCPB.

Group Number	Concentration (CO) ^a^, %	Cement Dosage (CD) ^b^, wt.%	AS Dosage (AD) ^d^, wt.%	FA/PCG	FA+PCG Content (FC) ^c^, wt.%	Activator Content (AC) ^e^, wt.%	Activator Type
Control	78%	3%	50	7/3	47	/	/
C-S1 ^f^					46	1	Na_2_SO_4_
C-S2					45	2	
C-S3					44	3	
C-S4					43	4	

^a^ CO: $\left(\frac{M_{AD}+M_{CD}+M_{FC}+M_{AC}}{M_{AD}+M_{CD}+M_{FC}+M_{AC}+M_{\mathrm{water}}}\times 100\%\right)$ ^b^ CD: $\frac{M_{CD}}{M_{AD}+M_{CD}+M_{FC}+M_{AC}}\times 100\%$. ^c^ FC: $\frac{M_{FA}+M_{PCG}}{M_{AD}+M_{CD}+M_{FA}+M_{PCG}+M_{AC}}\times 100\%$. ^d^ AD: $\frac{M_{AD}}{M_{AD}+M_{CD}+M_{FC}+M_{AC}}\times 100\%$. ^e^ AC: $\frac{M_{AC}}{M_{AD}+M_{CD}+M_{FC}+M_{AC}}\times 100\%$. ^f^ C-S1: represents a concentration of 78%, an AS dosage of 50%, a cement dosage of 3%, a Na_2_SO_4_ content of 1%, an FA and PCG content of 46%, and an FA/PCG ratio of 7/3.

**Table 3:** The statistical analysis of UCS results.

Sample	UCS, MPa			R, %	UCS, MPa			R,%	UCS, MPa			R,%	UCS, MPa			R, %
	3 d				7 d				14 d				28d			
	No. of Sample (*n*): 3				No. of Sample (*n*): 3				No. of Sample (*n*): 3				No. of Sample (*n*): 3			
	Mean	SD	CV		Mean	SD	CV		Mean	SD	CV		Mean	SD	CV	
Control	0.119	0.005	4.12	/	0.799	0.037	4.68	/	1.8	0.086	4.79	/	2.891	0.085	2.96	/
C-S1	0.376	0.015	3.99	215.97	2.438	0.052	2.13	205.13	2.924	0.109	3.72	62.44	3.4	0.075	2.22	17.61
C-S2	0.592	0.031	5.28	397.48	2.471	0.064	2.61	209.26	3.458	0.036	1.05	92.11	3.863	0.127	3.30	33.62
C-S3	0.545	0.008	1.44	357.98	2.057	0.038	1.83	157.45	3.284	0.066	2.02	82.44	4.312	0.095	2.20	49.15
C-S4	0.527	0.013	2.56	342.86	1.68	0.095	5.67	110.26	3.171	0.089	2.80	76.17	3.437	0.073	2.13	18.89

**Table 4:** Weight loss ratio of PCCPB with different Na_2_SO_4_ dosages.

Temperature Range, °C	Weight Change (3 d, 28 d), %				
	Control	C-S1	C-S2	C-S3	C-S4
I (30–250)	−2.25, −3.19	−3.11, −3.76	−3.45, −3.64	−3.3, −3.84	−3.31, −3.43
II (250–650)	−2.87, −1.06	−1.66, −1.47	−1.68, −1.34	−1.75, −1.24	−1.57, −2.72
III (650–750)	−0.80, −1.11	−0.31, −0.20	−0.19, −0.12	−0.22, −0.35	−0.16, −0.45
IV (750–900)	−0.42, −0.20	−0.04, −0.51	−0.03, −0.27	−0.03, −0.35	−0.06, −0.12

**Table 5:** Leaching results of ARFGB.

	Control, mg/L	C-S1, mg/L	C-S2, mg/L	C-S3, mg/L	C-S4, mg/L	Limit, mg/L
Mn	ND 1.2 × 10^−4^	ND 1.2 × 10^−4^	ND 1.2 × 10^−4^	ND 1.2 × 10^−4^	ND 1.2 × 10^−4^	0.1
Zn	ND 6.7 × 10^−4^	ND 6.7 × 10^−4^	ND 6.7 × 10^−4^	ND 6.7 × 10^−4^	ND 6.7 × 10^−4^	1
As	7.5 × 10^−3^	6.8 × 10^−3^	6.2 × 10^−3^	4.7 × 10^−3^	7.0 × 10^−3^	0.01
Cd	ND 5.0 × 10^−5^	ND 5.0 × 10^−5^	ND 5.0 × 10^−5^	ND 5.0 × 10^−5^	ND 5.0 × 10^−5^	0.005
Hg	1.8 × 10^−4^	1.4 × 10^−4^	1.1 × 10^−4^	8.1 × 10^−5^	1.3 × 10^−4^	0.001
Pb	ND 9.0 × 10^−5^	ND 9.0 × 10^−5^	ND 9.0 × 10^−5^	ND 9.0 × 10^−5^	ND 9.0 × 10^−5^	0.01
Cr	0.01	8.4 × 10^−3^	6.2 × 10^−3^	4.3 × 10^−3^	5.2 × 10^−3^	0.05
Cu	ND 8.0 × 10^−5^	ND 8.0 × 10^−5^	ND 8.0 × 10^−5^	ND 8.0 × 10^−5^	ND 8.0 × 10^−5^	1
Ba	ND 2.0 × 10^−4^	ND 2.0 × 10^−4^	ND 2.0 × 10^−4^	ND 2.0 × 10^−4^	ND 2.0 × 10^−4^	0.7
Ni	ND 6.0 × 10^−5^	ND 6.0 × 10^−5^	ND 6.0 × 10^−5^	ND 6.0 × 10^−5^	ND 6.0 × 10^−5^	0.02
Ag	ND 4.0 × 10^−5^	ND 4.0 × 10^−5^	ND 4.0 × 10^−5^	ND 4.0 × 10^−5^	ND 4.0 × 10^−5^	0.05
Se	2.3 × 10^−3^	1.8 × 10^−3^	1.5 × 10^−3^	1.1 × 10^−3^	1.6 × 10^−3^	0.01
Mo	0.02	0.017	0.014	0.012	0.013	0.07
Sb	1.9 × 10^−3^	1.6 × 10^−3^	1.4 × 10^−3^	1.1 × 10^−3^	1.3 × 10^−3^	0.005
Co	ND 3.0 × 10^−5^	1ND 3.0 × 10^−5^	ND 3.0 × 10^−5^	ND 3.0 × 10^−5^	ND 3.0 × 10^−5^	0.05

## Data Availability

The data used to support the findings of this study are included in the article.
